# Chemical Analysis of the Ingredients of 20% Aqueous Ethanol Extract of *Nardostachys jatamansi* through Phytochemical Study and Evaluation of Anti-Neuroinflammatory Component

**DOI:** 10.1155/2021/5901653

**Published:** 2021-04-22

**Authors:** Kwan-Woo Kim, Chi-Su Yoon, Sung-Joo Park, Gi-Sang Bae, Dong-Gu Kim, Youn-Chul Kim, Hyuncheol Oh

**Affiliations:** ^1^Institute of Pharmaceutical Research and Development, College of Pharmacy, Wonkwang University, Iksan 54538, Republic of Korea; ^2^Hanbang Cardio-Renal Syndrome Research Center Wonkwang University, Iksan 54538, Republic of Korea; ^3^Department of Herbal Crop Research, National Institute of Horticultural and Herbal Science, RDA, Eumseong 27709, Republic of Korea; ^4^Natural Medicine Research Center, Korea Research Institute of Bioscience and Biotechnology, Cheongju 28116, Republic of Korea; ^5^Department of Chemistry, University of Florida, Gainesville 32611, FL, USA; ^6^Department of Herbology, School of Korean Medicine, Wonkwang University, Iksan 54538, Republic of Korea; ^7^Department of Herbal Resources, Professional Graduate School of Oriental Medicine, Wonkwang University, Iksan 54538, Republic of Korea; ^8^Department of Pharmacology, School of Korean Medicine, Wonkwang University, Iksan 54538, Republic of Korea

## Abstract

*Nardostachys* spp. have been widely used in Asia as a folk medicine. In particular, the extracts of *Nardostachys jatamansi*, a species that grows in China, India, and Tibet, have been used to treat mental disorders, hyperlipidemia, hypertension, and convulsions. In this investigation, the potential of 20% aqueous ethanol extract of *N. jatamansi* (NJ20) as a botanical drug was explored by chemically investigating its constituents and its anti-neuroinflammatory effects on lipopolysaccharide- (LPS-) induced *in vitro* and *in vivo* models. Nine secondary metabolites were isolated and identified from NJ20, and quantitative analysis of these metabolites revealed desoxo-narchinol A as the major constituent. In LPS-challenged cells, pretreatment with NJ20 inhibited the LPS-induced excessive production of proinflammatory mediators, such as nitric oxide, prostaglandin E_2_, interleukin- (IL-) 1*β*, IL-6, and tumor necrosis factor-*α*. NJ20 also attenuated the overexpression of inducible nitric oxide synthase (iNOS) and cyclooxygenase-2. Additionally, pre-intraperitoneal injection of NJ20 downregulated the mRNA overexpression of IL-1*β*, IL-6, and iNOS in the prefrontal cortex, hypothalamus, and hippocampus of the LPS-stimulated C57BL/c mouse model. Chemical and biological investigations of NJ20 revealed that it is a potential inhibitor of LPS-induced neuroinflammatory responses in microglial cells and mouse models. The major active constituent of NJ20, desoxo-narchinol A, demonstrated anti-neuroinflammatory effects. Hence, our findings indicate that NJ20 may be a promising herbal mixture for developing a functional product and/or herbal drug for treating neuroinflammatory diseases.

## 1. Introduction

Microglial cells are important immune cells of the central nervous system (CNS) [1]. These cells are activated in response to brain injury, immunological stimuli, infection, and stress to sustain neuronal homeostasis. However, continuous stimulation leads to chronic and excessive microglial activation, and this results in the overproduction of different proinflammatory mediators, including nitric oxide (NO), prostaglandin E_2_ (PGE_2_), interleukin- (IL-) 1*β*, IL-6, and tumor necrosis factor- (TNF-) *α* [2]. Therefore, activated microglial cells can be regarded as a hallmark of neurodegenerative diseases. Accordingly, controlling the excessive production of pro-inflammatory mediators in activated microglial cells might be a method for treating and/or preventing of neuroinflammation and neurodegenerative diseases.

*Nardostachys jatamansi*, which belongs to the Valerianaceae family, is a plant indigenous to China, India, and Tibet. It is a perennial herb that grows to 10–60 cm in height [3], and has been traditionally used for the treatment of mental disorders, hyperlipidemia, hypertension, convulsions, digestive, neuropsychiatric disorders, epilepsy, hysteria, palpitations, and inflammation [4, 5]. According to Donguibogam, a traditional Korean medical manual published 400 years ago, the roots of *N. jatamansi* have sedative and analgesic properties for treating neurological symptoms and pain, respectively [6, 7]. *Nardostachys* spp. contain different secondary metabolites, such as terpenoids and lignans. Further, several aristolane-type and nardosinone-type sesquiterpenoids have been reported in *N. jatamansi* [8]. As revealed by pharmacological evaluations, sesquiterpenes present in the ethanol (EtOH) extract of *N. jatamansi* had a neuroprotective effect against amyloid beta peptide 42- (A*β*42-) induced neurotoxicity in SH-SY5Y neuroblastoma cells [6, 8]. Another report has demonstrated that the *N. jatamansi* extract increases insulin sensitivity in the skeletal muscles and suppresses glucose production in the liver, thus helping control hyperglycemia [3]. In another investigation, the water extract of *N. jatamansi* inhibited lipopolysaccharide- (LPS-) induced endotoxin shock by reducing proinflammatory cytokines via the inhibition of the mitogen-activated protein kinase (MAPK) pathways [9]. Recently, we reported the isolation of different components from the rhizomes and roots of *N. jatamansi*, including desoxo-narchinol A, narchinol B, nardosinone, isonardosinone, kanshone E, and kanshone B, as well as the beneficial effects of these compounds against LPS-induced neuroinflammation in BV2 microglial cells [10–13]. In the course of our continuous study on the bioactive components from *N. jatamansi*, C12-nardosinone type sesquiterpenoids, such as narchinol B and desoxo-narchinol A, were demonstrated to be the most potent anti-neuroinflammatory components in BV2 and primary microglial cells [10, 13].

Nowadays, botanical products account for significant portion of the drugs used worldwide in healthcare. As a result, there has been an increasing global interest in the investigation of botanical mixtures as drug products [14, 15]. It is often suggested that the long history of plant-based medicine would alleviate the need for early-stage scientific investigations in drug development, such as toxicology studies for human trials [15]. However, owing to the chemical and biological complexity of herbal natural extracts, characterizing and ensuring the pharmacological and quality consistency of botanical mixture remains challenging. In this perspective, this study was designed to assess the anti-neuroinflammatory effects of bioactivity-enriched 20% aqueous ethanol extract of *N. jatamansi* (NJ20) *in vitro* and *in vivo* and thereby determine its potential use as a botanical medicine. In addition, quantitative and qualitative analyses of the chemical constituents of NJ20 were conducted using spectroscopic and chromatographic methods.

## 2. Materials and Methods

### 2.1. Materials

1D nuclear magnetic resonance (NMR) spectra were obtained using in CDCl_3_ and CD_3_OD as solvents and a JNM ECP-400 spectrometer at 400 MHz for ^1^H NMR spectroscopy and at 100 MHz for ^13^C NMR spectroscopy (JEOL, Tokyo, Japan), using standard JEOL pulse sequences. Chemical shifts in the ^1^H and ^13^C NMR data were compared with the residual solvent peak (CDCl_3_: *δ*_H_/*δ*_C_ = 7.26/77.2; CD_3_OD: *δ*_H_/*δ*_C_ = 3.31/49.0). The electrospray ionization quadrupole time-of-flight mass spectroscopy (ESI Q-TOF MS/MS) system (AB SCRIEX Triple, USA) was used to obtain the ESI-MS data. High-performance liquid chromatography (HPLC) was performed using one of the following columns: prep-C_18_ column (21.2 × 150 mm; particle size, 5 *μ*m; flow rate, 5 mL/min); semi-prep-C_18_ column (10 × 250 mm; particle size, 5 *μ*m; flow rate, 3 mL/min); or semi-prep-silica gel column (10 × 250 mm; particle size, 5 *μ*m; flow rate, 3 mL/min). Reagent-grade solvents were used for the extractions and flash column chromatography (CC), and analytical-grade solvents were used for HPLC. Flash CC was performed using octadecyl-functionalized C_18_ silica gel (YMC, Kyoto, Japan) and silica gel (Merck, Kenilworth, NJ, USA).

### 2.2. Plant Materials, Extracts, and Isolation

*N. jatamansi* was obtained from a standard commercial source (Kwang Myung Dang, Ulsan, Korea) and was identified by CK Pharm Co., (Seoul, Korea). Voucher specimens (WK-2016-03) have been deposited at the College of Pharmacy Herbarium, Wonkwang University. The extraction and solvent partitioning methods for the isolation of compounds 1, 2, 6, and 9 were used as described in previous studies [11–13, 16]. To isolate the remaining components, the ethyl acetate (EtOAc) fraction of *N. jatamansi* (NJ(E), 21.8 g) was subjected to silica gel CC, using the hexane:EtOAc (3.5:1–1:0) and EtOAc:methanol (MeOH) (99:1–20 : 80) as the mobile phases, to yield 13 subfractions, NJ(E)-S1-S13. Subfraction NJ(E)-S2 was then subjected to C_18_ CC, with MeOH in H_2_O (50–100%) to yield seven subfractions. Subfraction NJ(E)-S2A was subjected to reverse phase medium pressure liquid chromatography (MPLC), with MeOH in H_2_O (50–100%) to yield 12 subfractions. Subfraction NJ(E)-S2AA6 was purified by reverse phase prep-HPLC, with acetonitrile in H_2_O (0.1% formic acid) and a gradient of 40–50% (0–20 min) to yield compound 5 (*t*_R_ = 15.2 min, 6.5 mg). Similarly, the CHCl_3_ fraction of the *N. jatamansi* (NJ(C), 6.3 g) was subjected to silica gel CC, with hexane:EtOAc (8:2–0:1) and EtOAc : MeOH (99:1–25 : 75) to yield 25 subfractions, NJ(C)-1-25. NJ(C)-7 was purified by normal phase semi prep-HPLC, with hexane in EtOH at a gradient of 97–75% (0–40 min) to yield compounds 7 (*t*_R_ = 19.1 min, 1.8 mg) and 8 (*t*_R_ = 18.3 min, 2.7 mg). NJ(C)-8 was then purified by normal phase semi prep-HPLC, with hexane in EtOH at a gradient of 92–90% (0–20 min) to yield compound 3 (*t*_R_ = 22.4 min, 0.6 mg).

To prepare desoxo-narchinol A-enriched NJ20, the dried rhizomes of *N. jatamansi* (500 g) were extracted with 20% (v/v) aqueous EtOH at 80°C, with using a reflux condenser system and evaporated using rotary evaporation to obtain NJ20 (87 g). A small amount of NJ20 was subjected to C_18_ CC, using MeOH in H_2_O (10–100%) as the mobile phase to yield six subfractions, NJ20-C1-6. The subfraction NJ20-C4 was further purified by using reverse phase semi-prep-HPLC, eluted using acetonitrile in H_2_O (0.1% formic acid) in a gradient method of 30–55% (0–20 min) to yield compound 4 (*t*_R_ = 18.2 min, 2.0 mg).

### 2.3. HPLC Profiling Methods

Chromatographic analysis was performed using an YL-9100 series HPLC instrument equipped with a sample injector and a photodiode array ultraviolet/visible (PDA-UV/Vis) detector (Young Lin, Korea). The analysis was conducted using Phenomenex Gemini NX-C18 column (4.6 mm × 250 mm; 5 *μ*m, Phenomenex Inc., CA, USA) as the stationary phase, with at injection volume of 20 *μ*L at room temperature. The mobile phase was composed of water (containing 0.1% formic acid) (A) and acetonitrile (B), with the following gradient system: 0–5 min linear change from 10% B to 10% B, 5–10 min linear change from 10% B to 20% B, 10–60 min linear change from 20% B to 40% B, 60–80 min linear change from 40% B to 70% B, 80–85 min linear change from 70% B to 100% B, and 85–90 min held at 100% B. The flow rate for HPLC analysis was set to 0.7 mL/min while the detection wavelength was set to 210 and 254 nm. Each sample was analyzed three times. Standard samples for HPLC were dissolved in 50% methanol, and NJ20 was prepared at a concentration of 10 mg/mL. For the quantitative analysis of compounds 1–9, standard compounds were prepared at concentrations between 0.0125 and 0.5 mg/mL, and calibration curves were constructed by plotting peak areas against the known concentrations of the standard compounds. The slope, intercept, and correlation coefficient (R) were calculated by linear regression analysis.

### 2.4. Chemicals and Reagents

Tissue culture reagents (RPMI1640, Dulbecco's modified Eagle's medium (DMEM), and fetal bovine serum (FBS)) were obtained from Gibco BRL Co. (Grand Island, NY, USA). LPS was purchased from Sigma-Aldrich (St. Louis, MO, USA). 3-[4,5-Dimethylthiazol-2-yl]-2, 5-diphenyltetrazolium bromide sodium (MTT) was obtained from Glentham Life Sciences (Corsham, UK). Anti-inducible nitric oxide synthase (iNOS) primary antibodies were purchased from Cayman (Ann Arbor, MI, USA) while anti-cyclooxygenase (COX)-2 and anti-*β*-actin antibodies were obtained from Santa Cruz Biotechnology (Dallas, TX, USA). Anti-mouse, anti-goat, and anti-rabbit secondary antibodies were purchased from Merck Millipore (Darmstadt, Germany).

### 2.5. BV2 Cell Culture and Isolation of Primary Microglial Cells

Immortalized murine BV2 microglial cells were maintained at 5 × 10^5^ cells/mL in 100 mm dishes in RPMI1640 supplemented with 10% (v/v) heat-inactivated FBS, penicillin G (100 units/mL), streptomycin (100 mg/mL), and l-glutamine (2 mM) and cultured at 37°C in a humidified atmosphere containing 5% CO_2_. The detailed protocol for the isolation of rat-derived primary microglial cells was described in our previous report [17]. The isolated primary microglial cells were seeded in 96-well plates (1 × 10^5^ cells/mL), 24-well plates (2 × 10^5^ cells/mL), and 6-well plates (5 × 10^5^ cells/mL) with B27® supplement (Thermo, Waltham, MA, USA) without insulin for 2–3 days; unattached cells were removed via washing prior to the experiments.

### 2.6. Animals

Female C57BL/6 mice (6-7 weeks old) weighing 15–20 g were purchased from the Orient Co., Ltd. (Seoul, Korea). Mice were bred and housed in standard shoebox cages in a climate-controlled room with an ambient temperature of 23 ± 2°C and a 12 h light-dark cycle for 3 days. All experiments were performed according to protocols approved by the Animal Care Committee of Wonkwang University and by the Institution Animal Care and Use Committee (IACUC) Certification of the Wonkwang University, Korea (WKU19-08). All efforts were made to minimize animal suffering, reduce the number of animals used, and utilize alternatives to *in vivo* technics, if available.

### 2.7. Animal Experiment Procedure

LPS (2 mg/kg, intraperitoneal (i.p.)), curcumin (50 mg/kg, i.p.), and NJ20 (10, and 50 mg/kg, i.p.) were dissolved in vehicle solution (1% dimethylsulfoxide (DMSO) with normal saline and phosphate-buffered saline (PBS)) prior to injection. Curcumin was used as the positive control to restore the LPS-induced alterations in brain inflammatory mediators based on previous study [18]. A total of 15 mice were used in the experiment and randomly assigned into control or experimental groups (*n* = 3 per group). Mice were then administered 2 mg/kg of LPS interperitoneally to induce systemic inflammation. In the control group, mice interperitoneally injected with the same volume of vehicle solution under the same conditions. In the LPS + curcumin group, 50 mg/kg of curcumin was injected once interperitoneally for 3 h prior to LPS (2 mg/kg) injection. In the LPS + NJ20 groups, 10 mg/kg or 50 mg/kg of NJ20 was administered interperitoneally for 3 h, prior to LPS injection (2 mg/kg). After 6 h of LPS injection, mice were sacrificed, and the fresh brain was removed and immersed in cold PBS. The prefrontal cortex, hypothalamus, and hippocampus were rapidly dissected and immediately stored at −80°C for polymerase chain reaction (PCR).

### 2.8. Cell Viability Assay

Cell viability was assessed using the MTT method. The detailed procedure for this assay has been described in our previous report [17]. BV2 and primary microglial cells were cultured in a 96-well plate at a density of 1 × 10^4^ cells/well and were then treated with NJ20 for 24 h. MTT solution (50 *μ*L) was added to each well at a final concentration of 0.5 mg/mL, and the cells were incubated for 3 h in a humidified incubator at 37°C. After removing the supernatant, the formed formazan was dissolved in 150 *μ*L of DMSO and mixed for 15 min. The optical density of the formazan formed in the untreated cells was considered to represent 100% viability, and the cell viability of the treated groups was expressed as a percentage of the viable cells obtained relative to that of controls. This assay was independently conducted three times.

### 2.9. Determination of Nitrite Concentration

The concentration of nitrite, an indicator of NO production, in the culture medium was estimated by the Griess method as described in our previous report [18]. BV2 and primary microglial cells were pre-treated with NJ20 for 3 h and stimulated with LPS (1 *μ*g/mL) for 24 h. An aliquot of each supernatant (100 mL) was mixed with the same volume of Griess reagent (0.1% (w/v) N-(1-naphthyl)-ethylenediamine and 1% (w/v) sulfanilamide in 5% (v/v) phosphoric acid) for 15 min at room temperature. The absorbance of the final product was measured spectrophotometrically at 540 nm using an enzyme-linked immunosorbent assay (ELISA) plate reader. Nitrite concentration in the samples was determined from a standard curve of sodium nitrite prepared in phenol red-free DMEM.

### 2.10. PGE_2_, IL-1*β*, IL-6, and TNF-*α* Assay

BV2 and primary microglial cells were pre-treated with NJ20 for 3 h and then stimulated with LPS (1 *μ*g/mL) for 24 h. The supernatant was collected to determine the concentrations of PGE_2_, IL-1*β*, IL-6, and TNF-*α* using commercially appropriate ELISA kits from ENZO Life Sciences (Farmingdale, NY, USA) and R&D Systems Inc. (Minneapolis, MN, USA). Three independent assays were performed according to the manufacturers' instructions.

### 2.11. Western Blot Analysis

The protocol used for western blot analysis has been previously reported [19]. Briefly, microglial cells were prepared using radioimmunoprecipitation assay buffer (Thermo Scientific). After the quantification and normalization of proteins by the Bradford protein assay (Bio-Rad Laboratories, CA, USA) to ensure equal amounts of loading, 30 *μ*g of protein from each sample was resolved using 7.5% and 12% sodium dodecyl sulfate-polyacrylamide gel electrophoresis. Then, proteins were electrophoretically transferred onto a Hybond enhanced chemiluminescence nitrocellulose membrane (Bio-Rad, Hercules). The membrane was then blocked with 5% skimmed milk and sequentially incubated with the primary antibodies (1 : 1000), and horseradish peroxidase-conjugated secondary antibodies (1 : 2000), followed by enhanced chemiluminescence detection (Amersham Pharmacia Biotech, Piscataway). The intensities of band signals were quantified using the densitometric ImageJ software (National Institutes of Health, Bethesda, MD). Data represent three independent experiments and were quantified by densitometric analysis.

### 2.12. RNA Sample Preparation and Quantitative Real-Time Polymerase Chain Reaction (qRT-PCR)

The brains were dissected to isolate the prefrontal cortex, hypothalamus, and hippocampus for qRT-PCR. The levels of the mRNA were analyzed based on our previous report [20]. Total RNA was isolated from cells using Trizol (Invitrogen, Carlsbad, CA), according to the manufacturer's recommendations, and spectrophotometrically quantified (at 260 nm). Total RNA (1 mg) was reverse-transcribed using the High Capacity RNA-to-cDNA kit (Applied Biosystems, Carlsbad, CA). Subsequently, the cDNA was amplified with SYBR Premix Ex Taq kit (TaKaRa Bio, Shiga, Japan) using a StepOnePlus Real-Time PCR system (Applied Biosystems, Foster City, CA). Each 20 mL of the reaction mixture contained 10 mL of SYBR Green PCR Master Mix, 0.8 mM of each primer, and diethyl pyrocarbonate (DEPC) treated water. The primer sequences were designed using PrimerQuest (Integrated DNA Technologies, Cambridge, MA). The sequences of the primers used in this experiment were as follows: IL-1*β*: forward (5′- AAT TGG TCA TAG CCC GCA CT -3′), reverse (5′- CTT CGT GGT CGT GTA ACG AA -3′); IL-6: forward (5′- ACT TCA CAA GTC GGA GGC TT -3′), reverse (5′ - TGT TGC TAC TAC GTG AAC GT -3′); iNOS: forward (5′ - GAC CAG ATA AGG CAA GCA C -3′), reverse (5′ -CTT GTC TTT GAC CCA GTA GC - 3′); and glyceraldehyde 3-phosphate dehydrogenase (GAPDH): forward (5′ - TTC ACC ACC ATG GAG AAG GC -3′), reverse (5′ -AGT ACT GGT GTC AGG TAC GG -3′). The optimal conditions for cDNA PCR amplification were established according to the manufacturer's instructions. The data were analyzed using StepOne software (Applied Biosystems, Foster City, CA) and the cycle number at the linear amplification threshold (C_t_) values for the endogenous control gene (GAPDH) and the target gene was recorded. Relative gene expression (target gene expression normalized to the expression of the endogenous control gene) was calculated using the comparative C_t_ method (2–^ΔΔCt^). The analysis was conducted three times, independently.

### 2.13. Statistical Analysis

Data are expressed as means ± standard deviations (SD) of at least three independent experiments. One-way analysis of variance and Tukey's multiple comparison tests were performed to compare three or more groups. GraphPad Prism software (version 3.03, GraphPad Software Inc, San Diego, CA, USA) was used for statistical analyses.

## 3. Results and Discussion

### 3.1. HPLC Chemical Profiling and Quantification of NJ20 Components

**Figure 1:**
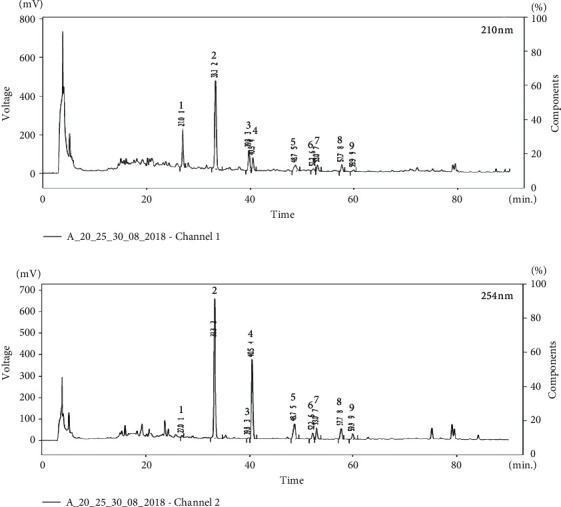
High-performance liquid chromatography (HPLC) chromatography of NJ20 at 210 and 254 nm. The HPLC method is described in the Materials and Methods section.

**Figure 2:**
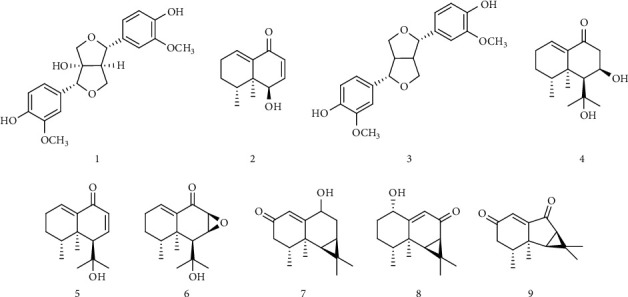
Chemical structure of the nine metabolites in NJ20 (1: 8*α*–hydroxypinoresinol, 2: desoxo-narchinol A 3: pinoresinol, 4: nardosinonediol, 5: kanshone A 6: isonardosinone, 7: deblion, 8: 1*α*-hydroxy-(-)-aristolone, and 9: nardoaristolone B).

The chemical profiles of *Nardostachys* spp. have been abundantly reported. For instance, phenolic compounds such as protocatechuic acid and syringic acid were identified by HPLC analysis of the methanolic extract of *N. jatamansi* [21]. A gas chromatography (GC)/MS analysis of the essential oils of *N. chinensis* also revealed that this species contains guaiane- and aristolane-type sesquiterpenoids [22]. Furthermore, recently, an optimized HPLC-UV method for the quantitative analysis of sesquiterpenes in *Nardostachys* Radix et Rhizoma, including optimized sample preparation conditions, was reported. Results of this method suggested that 20% (v/v) aqueous EtOH extract of *N. jatamansi* contained the highest amount of desoxo-narchinol A among the extracts analyzed [23]. Because desoxo-narchinol A was identified as one of the most potent anti-neuroinflammatory components of the MeOH extract of *N. jatamansi* in our previous study, the findings of Le et al. led us to postulate that the extraction of *N. jatamansi* with 20% aqueous EtOH would provide a bioactive extract with potent anti-inflammatory effects. Accordingly, we aimed to assess the composition of NJ20 to explore its potential as an herbal medicine and to support its quality and therapeutic consistency. In the current study, nine metabolites from NJ20 were identified as 8*α*–hydroxypinoresinol (1) [16], desoxo-narchinol A (2) [13], pinoresinol (3) [24], nardosinonediol (4) [25], kanshone A (5) [25], isonardosinone (6) [11], deblion (7) [25], 1*α*-hydroxy-(-)-aristolone (8) [26], and nardoaristolone B (9) [12], by comparing their retention time in HPLC chromatograms and their corresponding structures determined using NMR spectroscopy with previous findings (Figures 1 and 2). The quantitative analysis of compounds 1–9 in NJ20 was performed by HPLC. The contents of each compound in the extract were calculated from the peak areas by interpolation to standard calibration curves (Table 1). In this analysis, NJ20 was found to contain two furofuran-lignans, compounds 1 and 3 at 0.38 ± 0.01% and 0.30 ± 0.03%, respectively. Previously, compound 1 had been reported to ameliorate cerulin-induced acute pancreatitis by inhibiting nuclear factor kappa B (NF-*κ*B) activation in a mouse model [16]. Reportedly, compound 3 has mild anti-inflammatory effects in LPS-induced RAW264.7 cell lines [24]. Furthermore, NJ20 was found to contain four nardosinone-type sesquiterpenes, compounds 2, 4, 5, and 6 at 5.76 ± 0.15%, 1.54 ± 0.06%, 0.76 ± 0.04%, and 0.92 ± 0.15%, respectively. As expected and consistent with a previous report [23], the unusual 12 carbon-containing nardosinone-type sesquiterpene, desoxo-narchinol A (2), was identified as the major component in NJ20, suggesting that the biological effects of NJ20 are mainly derived from this compound. Several studies have reported that desoxo-narchinol A exhibits different biological effects such as anti-inflammatory effects in peritoneal macrophages and an endotoxin shock model [27], anti-neuroinflammatory effects in BV2 microglial cells [13], and acute pancreatitis reduction [28]. Furthermore, we identified NJ20 to contain three aristolone-type sesquiterpenes, compounds 7, 8, and 9 at 0.32 ± 0.03%, 0.62 ± 0.04%, and 0.20 ± 0.02% respectively. Compound 9 is a rather unusual aristolone-type sesquiterpene with 14 carbons, which has been reported to have anti-neuroinflammatory effects [12].

### 3.2. Effect of NJ20 on the Production of Proinflammatory Mediators in LPS-Induced BV2 and Primary Microglial Cells

iNOS and COX-2 are important inducible enzymes in the regulation of inflammatory responses [29], participating in the production of the pro-inflammatory mediators, NO and PGE_2_, respectively [30]. The sustained overproduction of NO and PGE_2_ by the upregulation of iNOS and COX-2 protein expression is responsible for the development of inflammatory diseases, including stroke, osteoarthritis, rheumatoid arthritis, and spinal cord injury [31]. In addition, several pro-inflammatory cytokines, such as IL-1*β*, IL-6, and TNF-*α*, are known to modulate immune responses and inflammation [32]. External stimuli, including LPS and interferon gamma (IFN-*γ*), could induce excessive activation of microglia and increase the aforementioned pro-inflammatory cytokines, leading to exacerbation of neuronal injury and neuroinflammatory responses [33,34]. Therefore, we initially examined the suppressive effect of NJ20 against LPS-induced release of pro-inflammatory mediators in BV2 microglial cells.

**Figure 3:**
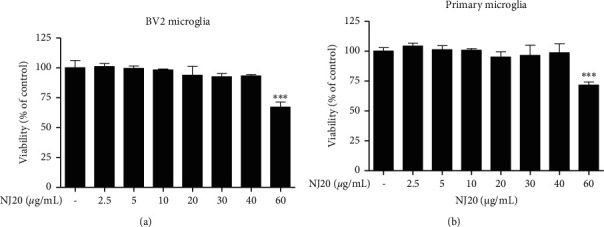
The effect of NJ20 on BV2 (a) and primary microglial cell (b) viability. Cells were treated with NJ20 at the indicated concentrations for 24 h and cell viability was determined by MTT assay. Values are presented as mean ± SD of three independent experiments. ^*∗∗∗*^*p* < 0.001 compared with the control group.

**Figure 4:**
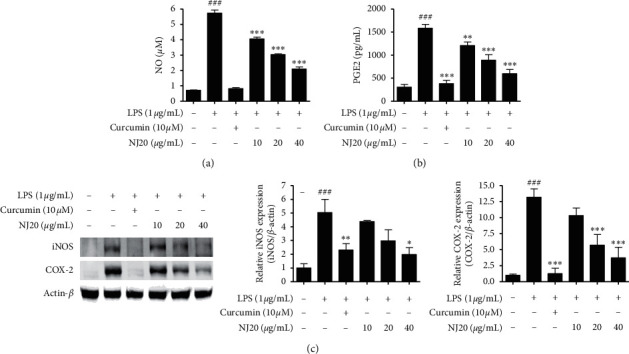
Effects of NJ20 on LPS-induced production of nitrite and PGE_2_, and protein expression of iNOS and COX-2 in BV2 microglial cells. Cells were pre-treated with or without the indicated concentration of NJ20 for 3 h and stimulated with LPS (1 *μ*g/mL) for 24 h. ((a), (b)) The collected supernatants were used to determine nitrite and PGE_2_ levels using the Griess reaction and appropriate ELISA kits. Values are presented as mean ± SD of three independent experiments. (c) The collected cells were lysed, and the lysates were then used to determine the expression of iNOS and COX-2 proteins by western blot analysis. Representative blots from three independent experiments are shown. Curcumin (10 *μ*M) was used as the positive control. ^###^*p* < 0.001 compared with the control group. ^*∗*^*p* < 0.05, ^∗∗^*p* < 0.05, and ^*∗∗∗*^*p* < 0.001 compared with the LPS-treated group.

**Figure 5:**
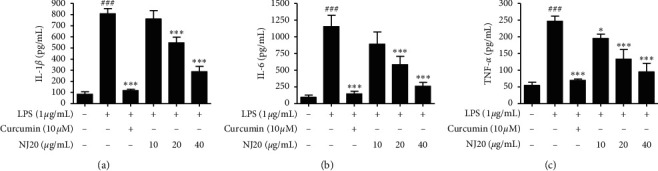
Effects of NJ20 on LPS-induced production of pro-inflammatory cytokines in BV2 microglial cells. Cells were pre-treated with or without the indicated concentration of NJ20 for 3 h and stimulated with LPS (1 *μ*g/mL) for 24 h. The concentration of cytokines was measured by ELISA. Values are expressed as mean ± SD of three independent experiments. Curcumin (10 *μ*M) was used as the positive control. ^###^*p* < 0.001 compared with the control group. ^*∗*^*p* < 0.05 and ^*∗∗∗*^*p* < 0.001 compared with the LPS-treated group.

To ensure that the anti-neuroinflammatory effects were not induced by the cytotoxicity of NJ20, the cytotoxic concentration of NJ20 was evaluated using the MTT method. BV2 cells were treated with NJ20 ranging from 2.5 *μ*g/mL to 60 *μ*g/mL for 24 h. Although toxicity was not detected in the concentration range of 2.5–40 *μ*g/mL, the cell survival rate was reduced by ∼33% in the 60 *μ*g/mL NJ20-treated group compared to the control group (Figure 3(a)). Therefore, further experiments were performed with 40 *μ*g/mL as the highest concentration of NJ20. Subsequently, the levels of NO, PGE_2_, iNOS, COX-2, IL-1*β*, IL-6, and TNF-*α*, in the cells were analyzed by Griess reaction, ELISA, and western blot analysis. Stimulation of BV2 cells with LPS led to a significant increase in the production of NO and PGE_2_, i.e., up to 8.2-fold, relative to that of the unstimulated cells. However, pre-treatment with NJ20 inhibited LPS-induced NO (Figure 4(a)) and PGE_2_ overproduction (Figure 4(b)) in a dose-dependent manner. At the 40 *μ*g/mL concentration, the excessive production of NO and PGE_2_ was inhibited by 72.4% and 77.3%, respectively. In addition, the densitometric analysis revealed that LPS stimulation upregulated the expression of iNOS and COX-2 in the cells by 5.0- and 13.2-fold, respectively, whereas pre-treating the cells with NJ20 reduced the overexpression of both enzymes (Figure 4(c)). Moreover, at the 40 *μ*g/mL concentration, the expression of iNOS and COX-2 proteins was similar to that observed in the control group. Levels of pro-inflammatory cytokines, including IL-1*β*, IL-6, and TNF-*α*, in LPS-induced BV2 microglial cells were evaluated using an ELISA kit. Following LPS stimulation, IL-1*β*, IL-6, and TNF-*α* levels increased by 9.5, 12.2-, and 4.5-fold, respectively, relative to those following no stimulation. Pretreatment with NJ20 reduced the overproduction of these cytokines in a dose-dependent manner, and the inhibitory percentages were 72.1%, 84.4%, and 78.9%, respectively, at the 40 *μ*g/mL concentration (Figure 5).

**Figure 6:**
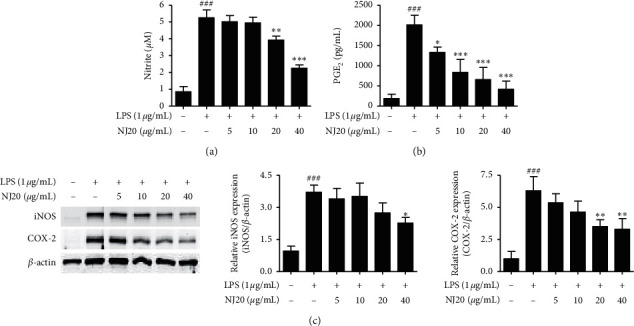
Effects of NJ20 on LPS-induced production of nitrite and PGE_2_, and the protein expression of iNOS and COX-2 in primary microglial cells. Cells were pre-treated with or without the indicated concentration of NJ20 for 3 h and stimulated with LPS (1 *μ*g/mL) for 24 h. ((a), (b)) The collected supernatants were used to determine nitrite and PGE_2_ levels using the Griess reaction and appropriate ELISA kits. Values are presented as mean ± SD of three independent experiments. (c) The collected cells were lysed, and the lysates were then used to determine the expression of iNOS and COX-2 proteins by western blot analysis. Representative blots from three independent experiments are shown. ^###^*p* < 0.001 compared with the control group. ^*∗*^*p* < 0.05, ^∗∗^*p* < 0.01, and ^*∗∗∗*^*p* < 0.001 compared with the LPS group.

**Figure 7:**
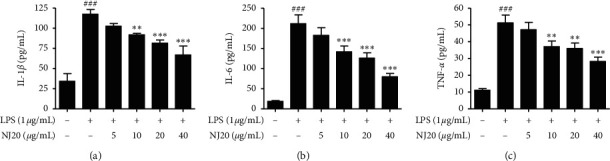
Effects of NJ20 on LPS-induced production of pro-inflammatory cytokines in primary microglial cells. Cells were pre-treated with or without the indicated concentration of NJ20 for 3 h and stimulated with LPS (1 *μ*g/mL) for 24 h. The concentration of cytokines was measured by ELISA. Values are expressed as mean ± SD of three independent experiments. ^###^*p* < 0.001 compared with the control group. ^∗∗^*p* < 0.01 and ^*∗∗∗*^*p* < 0.001 compared with the LPS-treated group.

Although BV2 cells are frequently employed as an *in vitro* model for research on neurodegenerative diseases, some disparities exist between the immortalized BV2 cell line and activated microglia *in vivo* [35]. Because cultured primary microglial cells are suggested to be a better *in vivo* disease model, we examined the neuroinflammatory effects of NJ20 in primary microglial cells derived from the cerebral cortices or substantia nigra of a rat early after birth. Overall, the neuroinflammatory effects of NJ20 in the primary microglial cells were almost analogous to those in BV2 cells (Table 2). Further, toxicity was not identified in the dose range of 2.5–40 *μ*g/mL of NJ20 in primary microglial cells, and cell viability was approximately 71.6% at 60 *μ*g/mL (Figure 3(b)). Compared with untreated cells, LPS-treated cells showed 6.2- and 10.6-fold increase in the levels of NO and PGE_2_, respectively. However, the overproduction of NO and PGE_2_ was suppressed by pretreatment with NJ20 in a concentration-dependent manner, with a magnitude of suppression being 68.2%, and 87.6%, respectively (Figures 6(a) and 6(b)) at 40 *μ*g/mL of NJ20. These inhibitory effects correlated with the suppressive effects of NJ20 against the overexpression of iNOS and COX-2 proteins in LPS-stimulated primary microglial cells (Figure 6(c)), which were upregulated by 3.9- and 6.3-fold, respectively. ELISA revealed that the production of the proinflammatory cytokines, IL-1*β*, IL-6, and TNF-*α*, was upregulated by LPS stimulation by 3.4-, 11.3-, and 4.6-fold, respectively, relative to the untreated cells. However, this response was attenuated by pre-treatment with NJ20, demonstrating inhibitory rates of 60.7%, 57.3%, and 68.4%, respectively, with 40 *μ*g/mL of NJ20 (Figure 7).

Desoxo-narchinol A is a nardosinone-type sesquiterpenoid isolated from the rhizomes and roots of *N. jatamansi*. Recently, we reported that this compound significantly inhibited the overproduction of NO, PGE_2_, IL-1*β*, IL-6, and TNF-*α* in LPS-induced BV2 and primary microglial cells, with IC_50_ of 2–6 *μ*M for each proinflammatory mediator [13]. In the current investigation, microglial cells were pretreated with the highest noncytotoxic concentration of NJ20 (40 *μ*g/mL) to detect significant anti-neuroinflammatory effects. Considering the determined content of desoxo-narchinol A in NJ20 (5.76 ± 0.15%) and the molecular weight of this compound, the highest concentration of NJ20 employed in this study was estimated as 12.0 ± 0.3 *μ*M, which was similar to the highest concentration used in our previous study [13]. Additionally, the IC_50_ of NJ20 against the upregulation of proinflammatory mediators and cytokines (Table 2) was consistent with the converted IC_50_, which can be calculated from the content of desoxo-narchinol A in NJ20 and the IC_50_ of the pure compound described in our previous report [13]. Therefore, it was postulated that the anti-neuroinflammatory effects of NJ20 were mainly derived from desoxo-narchinol A in this botanical mixture. In our previous study that aimed to elucidate the underlying mechanism, desoxo-narchinol A was demonstrated to inhibit the LPS-induced activation of the NF-*κ*B pathway in both BV2 and primary microglial cells through heme oxygenase (HO)-1/nuclear transcription factor erythroid-2-related factor 2 (Nrf2) signaling pathway, leading to the suppressed release of proinflammatory mediators [10]. Because desoxo-narchinol A was likely responsible for the anti-neuroinflammatory effects of NJ20, the anti-neuroinflammatory effects of NJ20 would be mediated through analogous pathways.

### 3.3. Effect of NJ20 on the mRNA Level of Proinflammatory Mediators in LPS-Induced Mice

Microglial cells are broadly distributed in large nonoverlapping regions throughout the brain and spinal cord and account for 20% of the total glial cell population within the CNS parenchyma, including the prefrontal cortex, hypothalamus, and hippocampus [36]. The prefrontal cortex is associated with motor function, emotional or social behavior, and temporal memory [37]. The hypothalamus can regulate energy balance by controlling food intake, energy storage, and energy expenditure. The inflammatory responses in the hypothalamus are associated with energy balance disruption, leading to the development of obesity [38]. The hippocampus is the brain region responsible for learning and memory, and it is damaged in patients with neurodegenerative diseases, leading to cognitive impairment [39]. These aforementioned brain tissues are frequently used to investigate microglial activation and, as demonstrated, increase in the expression of ionized calcium-binding adapter molecule 1 (Iba-1), a well-known marker of activated microglia and macrophages, is associated with inflammation [40–42]. Additionally, microglial activation was reported to induce peripheral inflammation, and systemic injection of LPS resulted in increased mRNA levels of pro-inflammatory cytokines in the prefrontal cortex, hypothalamus, and hippocampus [43].

**Figure 8:**
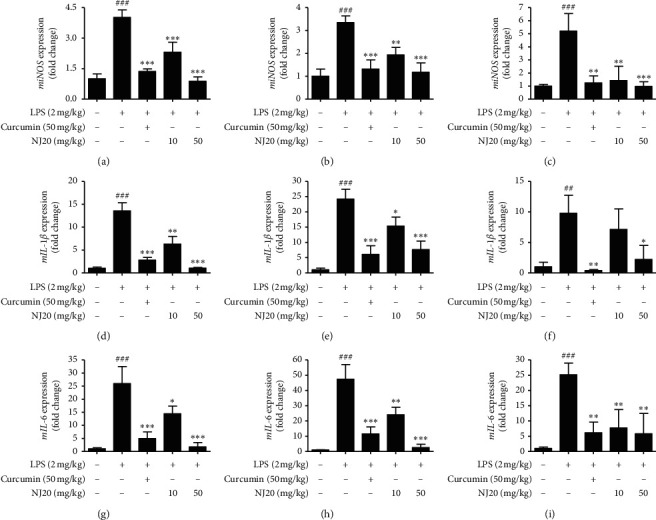
Effects of NJ20 on LPS-induced mRNA expression of iNOS (a, b, c), IL-1*β* (d, e, f), and IL-6 (g, h, i) in the prefrontal cortex, hypothalamus, and hippocampus in the brain of C57BL/c mice. C57BL/c mice were pre-treated with 10 and 50 mg/kg of NJ20 for 3 h followed by LPS (2 mg/kg) for 6 h. Curcumin (50 mg/kg) was used as the positive control. Values are expressed as mean ± SD. ^##^*p* < 0.001, and ^###^*p* < 0.001 compared with the control group. ^*∗*^*p* < 0.05, ^∗∗^*p* < 0.01, and ^*∗∗∗*^*p* < 0.001 compared with the LPS-treated group.

The anti-neuroinflammatory effects of NJ20 observed in the two LPS-stimulated microglial cellular models prompted us to examine these effects in LPS-treated *in vivo* models to gain a better insight into the therapeutic potential of NJ20 in diseases associated with neuroinflammatory responses. In the present investigation, C57BL/6 mice were pretreated with 10 mg/kg and 50 mg/kg of NJ20 for 3 h followed by a single intraperitoneal administration of 2 mg/kg of LPS for 6 h to induce acute systemic inflammation. Generally, the pro-inflammatory mediators released in the periphery do not diffuse across the blood-brain barrier (BBB). However, it is well documented that peripheral injection with LPS can transfer the signal to the brain and induce different central effects mediated by pro-inflammatory mediators released from the microglia [44,45]. Numerous studies have demonstrated that multiple peripheral injection of LPS to mice increases the number of cluster of differentiation (CD)68-, Iba-1-, F4/80-, CD11-, or CD45-positive cells, which are markers of microglial activation [46–48]. However, in mice, a single administration of LPS was sufficient to induce a neuroinflammatory response [49, 50], despite other reports revealing that microglial activation was not observed after single LPS administration. The discrepancies regarding the differences in LPS challenge conditions are attributed to different experimental details, such as the quality and quantity of LPS applied, mouse strain used, site of injection, or sacrifice time after LPS stimulation [44]. Curcumin (50 mg/kg), which is known to reduce inflammatory response in the brain, was employed as a positive control [18, 51]. After treatment, we dissected the prefrontal cortex, hypothalamus, and hippocampus from mice brains and evaluated the effects of NJ20 on the mRNA expression of the proinflammatory enzyme iNOS and pro-inflammatory cytokines IL-1*β* and IL-6 in three dissected brain tissues using qRT-PCR (Figure 8). Initially, the mRNA level of iNOS in the prefrontal cortex, hypothalamus, and hippocampus of the LPS-treated group was significantly increased compared with that of the untreated group. However, pre-treatment with 10 mg/kg and 50 mg/kg of NJ20 significantly decreased the LPS-induced iNOS mRNA level in all three brain regions (Figures 8(a), 8(b), and 8(c)). The inhibitory effect of NJ20 occurred in a dose-dependent manner, and its potency was comparable to that of the positive control, curcumin. We also determined the mRNA expression level of the pro-inflammatory cytokines, IL-1*β* and IL-6, in the prefrontal cortex, hypothalamus, and hippocampus 6 h after LPS injection. Intraperitoneal injection with 2 mg/kg of LPS elevated the mRNA levels of IL-1*β* and IL-6 in all brain tissues. However, pre-treatment with both 10 mg/kg and 50 mg/kg of NJ20 significantly inhibited the elevation of IL-1*β* mRNA levels in the prefrontal cortex and hypothalamus (Figures 8(d) and 8(e)), whereas only 50 mg/kg of NJ20 could decrease this response in the hippocampus region (Figure 8(f)). In contrast, the elevation of the mRNA levels of IL-6 was attenuated by pre-treatment with NJ20 in all three brain regions (Figures 8(g), 8(h), and 8(i)). Pretreatment with 50 mg/kg of curcumin markedly inhibited LPS-induced mRNA expression of IL-1*β* and IL-6 in the prefrontal cortex, hypothalamus, and hippocampus, which resulted in effects similar to those mediated by 50 mg/kg of NJ20. Taken together, our findings clearly demonstrate that the anti-neuroinflammatory effects of NJ20 observed in the two different microglial cellular models are well correlated with its suppressive effects against LPS-induced neuroinflammation *in vivo*.

## 4. Conclusion

In the current study, we performed qualitative and quantitative analyses of the secondary metabolites and anti-neuroinflammatory effects of NJ20 using three experimental models, BV2 and primary microglial cells, and a mouse model of LPS-induced neuroinflammation. Based on our findings, we concluded that NJ20 contains several metabolites, including 8*α*–hydroxypinoresinol (1), desoxo-narchinol A (2), pinoresinol (3), nardosinonediol (4), kanshone A (5), isonardosinone (6), deblion (7), 1*α*-hydroxy-(-)-aristolone (8), and nardoaristolone B (9). Pretreatment with NJ20 attenuated the production of proinflammatory mediators, such as NO, PGE_2_, IL-1*β*, IL-6, and TNF-*α*, and the protein expression of iNOS and COX-2 in LPS-induced BV2 and primary microglial cells. Although some studies have revealed that the BV2 cell line may not always represent the primary microglial cells involved in inflammation [52], consistent anti-neuroinflammatory effects of NJ20 were observed in both cellular models. In a further evaluation of the anti-neuroinflammatory effects of NJ20 using an *in vivo* model, NJ20 was found to decrease the mRNA levels of iNOS, IL-1*β*, and IL-6 in the prefrontal cortex, hypothalamus, and hippocampus of LPS-administered mice. Considering the potency of desoxo-narchinol A in the cellular models, the quantitative analysis of NJ20 suggested that the presence of desoxo-narchinol A is chiefly responsible for the anti-neuroinflammatory potency of NJ20 observed in the cellular models. Traditionally, *N. jatamansi* was used as a medicinal herb to treat different pathological conditions, including neurological and inflammatory diseases. Based on this use and its chemical constituents, the present and our previous studies regarding the anti-neuroinflammatory secondary metabolites of *N. jatamansi* provide scientific support for the traditional use of this medicinal plant. Further pharmacological investigations on desoxo-narchinol A-enriched extracts from this plant such as the NJ20 would potentiate the development of botanical drugs for the treatment of diseases involving abnormal neuroinflammatory responses.

## Figures and Tables

**Table 1:** Quantitative HPLC analysis of compounds 1–9 in NJ20.

No.	Analytes	Detection wavelength (nm)	Retention time (min)	Regression equation	*r* ^2^	Content (g/g ^*∗*^ 100) (%)
1	8*α*–Hydroxypinoresinol	210	26.82	*y* = 3240.6*x* + 973.28	0.9888	0.38 ± 0.01
2	Desoxo-narchinol A	254	33.22	*y* = 1114.1*x* + 324.93	0.9999	5.76 ± 0.15
3	Pinoresinol	210	39.48	*y* = 1131.2*x* + 1354.5	0.9933	0.30 ± 0.03
4	Nardosinonediol	254	40.52	*y* = 2205.3*x* + 545.52	0.9657	1.54 ± 0.06
5	Kanshone A	254	48.76	*y* = 1324.2*x* + 170.38	1.0000	0.76 ± 0.04
6	Isonardosinone	254	51.97	*y* = 386.52*x* + 46.90	1.0000	0.92 ± 0.15
7	Deblion	254	53.08	*y* = 1786.6*x* + 91.597	0.9999	0.32 ± 0.03
8	1*α*-Hydroxy-(-)-aristolone	254	57.73	*y* = 982.07*x* + 67.45	0.9983	0.62 ± 0.04
9	Nardoaristolone B	254	59.38	*y* = 1720.3*x* + 45.37	0.9998	0.20 ± 0.02

**Table 2:** Inhibitory effects of NJ20 on LPS-induced pro-inflammatory mediators and cytokines in BV2 and primary microglial cells.

Proinflammatory mediators and cytokines	BV2 microglial cells IC50 (*μ*g/mL)	Primary microglial cells IC50 (*μ*g/mL)
NO	12.57 ± 0.10	28.83 ± 1.08
PGE2	15.36 ± 2.76	7.14 ± 0.71
IL-1*β*	12.69 ± 1.06	25.62 ± 0.93
IL-6	14.15 ± 0.66	21.12 ± 2.53
TNF-*α*	13.52 ± 3.20	29.90 ± 6.77

## Data Availability

The datasets generated and analyzed during the current study are available from the corresponding author upon reasonable request.

## Abbreviations

A*β*42: Amyloid beta peptide 42
BBB: Blood-brain barrier
CC: Column chromatography
CD: Cluster of differentiation
CNS: Central nervous system
COX-2: Cyclooxygenase-2
DMEM: Dulbecco's modified Eagles' medium
DMSO: Dimethyl sulfoxide
ELISA: Enzyme-linked immunosorbent assay
ESI Q-TOF MS/MS: Electrospray ionization quadrupole time-of-flight mass spectroscopy
FBS: Fetal bovine serum
GAPDH: Glyceraldehyde 3-phosphate dehydrogenase
GC: Gas chromatography
HO-1: Heme oxygenase-1
HPLC: High-performance liquid chromatography
i.p.: Intraperitoneal
Iba-1: Ionized calcium-binding adapter molecule 1
IL: Interleukin
iNOS: Inducible nitric oxide synthase
LPS: Lipopolysaccharide
MAPK: Mitogen-activated protein kinase
MPLC: Medium pressure liquid chromatography
MTT: 3-[4,5-Dimethylthiazol-2-yl]-2, 5-diphenyltetrazolium bromide sodium
NF-*κ*B: Nuclear factor kappa B
NMR: Nuclear magnetic resonance
NO: Nitric oxide
Nrf2: Nuclear transcription factor erythroid-2-related factor 2
PBS: Phosphate-buffered saline
PCR: Poly chain reaction
PDA-UV/Vis: Photodiode array ultraviolet/visible
PGE_2_: Prostaglandin E2
SD: Standard derivation
TNF: Tumor necrosis factor.

## Supplementary Materials

Supplementary Materials Figure S1: ^1^H-NMR spectrum of compound 1. Figure S2: ^13^C-NMR spectrum of compound 1. Figure S3: HPLC quantitative data of compound 1. Figure S4: ^1^H-NMR spectrum of compound 2. Figure S5: ^13^C-NMR spectrum of compound 2. Figure S6: HPLC quantitative data of compound 2. Figure S7: ^1^H-NMR spectrum of compound 3. Figure S8: ^13^C-NMR spectrum of compound 3. Figure S9: HPLC quantitative data of compound 3. Figure S10: ^1^H-NMR spectrum of compound 4. Figure S11: ^13^C-NMR spectrum of compound 4. Figure S12: HPLC quantitative data of compound 4. Figure S13: ^1^H-NMR spectrum of compound 5. Figure S14. ^13^C-NMR spectrum of compound 5. Figure S15: HPLC quantitative data of compound 5. Figure S16: ^1^H-NMR spectrum of compound 6. Figure S17: ^13^C-NMR spectrum of compound 6. Figure S18: HPLC quantitative data of compound 6. Figure S19: ^1^H-NMR spectrum of compound 7. Figure S20: ^13^C-NMR spectrum of compound 7. Figure S21: HPLC quantitative data of compound 7. Figure S22: ^1^H-NMR spectrum of compound 8. Figure S23: HPLC quantitative data of compound 8. Figure S24: ^1^H-NMR spectrum of compound 9. Figure S25: ^13^C-NMR spectrum of compound 9. Figure S26: HPLC quantitative data of compound 9. Figure S27: HPLC chromatograms of NJ20, compounds 1, 2, 3 and 4. Figure S28: HPLC chromatograms of NJ20, compounds 5, 6, 7, 8 and 9.
