# Supplementary material for: Chemical Analysis of the Ingredients of 20% Aqueous Ethanol Extract of Nardostachys jatamansi through Phytochemical Study and Evaluation of Anti-Neuroinflammatory Component
Source: Evid Based Complement Alternat Med. 2021 Apr 22;2021:5901653. doi: 10.1155/2021/5901653 (PMC8084687; doi:10.1155/2021/5901653)
Supplement: Supplementary Materials — Figure S1: 1H-NMR spectrum of compound 1. Figure S2: 13C-NMR spectrum of compound 1. Figure S3: HPLC quantitative data of compound 1. Figure S4: 1H-NMR spectrum of compound 2. Figure S5: 13C-NMR spectrum of compound 2. Figure S6: HPLC quantitative data of compound 2. Figure S7: 1H-NMR spectrum of compound 3. Figure S8: 13C-NMR spectrum of compound 3. Figure S9: HPLC quantitative data of compound 3. Figure S10: 1H-NMR spectrum of compound 4. Figure S11: 13C-NMR spectrum of compound 4. Figure S12: HPLC quantitative data of compound 4. Figure S13: 1H-NMR spectrum of compound 5. Figure S14. 13C-NMR spectrum of compound 5. Figure S15: HPLC quantitative data of compound 5. Figure S16: 1H-NMR spectrum of compound 6. Figure S17: 13C-NMR spectrum of compound 6. Figure S18: HPLC quantitative data of compound 6. Figure S19: 1H-NMR spectrum of compound 7. Figure S20: 13C-NMR spectrum of compound 7. Figure S21: HPLC quantitative data of compound 7. Figure S22: 1H-NMR spectrum of compound 8. Figure S23: HPLC quantitative data of compound 8. Figure S24: 1H-NMR spectrum of compound 9. Figure S25: 13C-NMR spectrum of compound 9. Figure S26: HPLC quantitative data of compound 9. Figure S27: HPLC chromatograms of NJ20, compounds 1, 2, 3 and 4. Figure S28: HPLC chromatograms of NJ20, compounds 5, 6, 7, 8 and 9. [file 5901653.f1.docx]

**Supplementary data**

**Chemical analysis of the ingredients of 20% aqueous ethanol extract of *Nardostachys jatamansi* through phytochemical study and evaluation of anti-neuroinflammatory component**

Kwan-Woo Kim^1,2,3,a^, Chi-Su Yoon^1,2,4,5,a^, Sung-Joo Park^2,6,7^, Gi-Sang Bae^2,8^, Dong-Gu Kim^2^, Youn-Chul Kim^1,2^, and Hyuncheol Oh^1,2,*^

*^1^Institute of Pharmaceutical Research and Development, College of Pharmacy, Wonkwang University, Iksan, 54538, Republic of Korea*

*^2^Hanbang Cardio-Renal Syndrome Research Center Wonkwang University, Iksan, 54538, Republic of Korea*

*^3^Department of Herbal Crop Research, National Institute of Horticultural and Herbal Science, RDA, Eumseong, 27709, Republic of Korea*

*^4^Natural Medicine Research Center, Korea Research Institute of Bioscience and Biotechnology, Cheongju 28116, Republic of Korea*

*^5^Department of Chemistry, University of Florida, Gainesville, FL 32611, USA*

*^6^Department of Herbology, School of Korean Medicine, Wonkwang University, Iksan, 54538, Republic of Korea*

*^7^Department of Herbal Resources, Professional Graduate School of Oriental Medicine, Wonkwang University, Iksan, 54538, Republic of Korea*

*^8^Department of Pharmacology, School of Korean Medicine, Wonkwang University, Iksan, 54538, Republic of Korea*

**List of Supplementary Figures**

**Page**

**Figure S1**. ^1^H NMR spectrum of compound **1 S4**

**Figure S2**. ^13^C NMR spectrum of compound **1 S5**

**Figure S3**. HPLC quantitative data of compound **1 S6**

**Figure S4**. ^1^H NMR spectrum of compound **2 S7**

**Figure S5**. ^13^C NMR spectrum of compound **2 S8**

**Figure S6**. HPLC quantitative data of compound **2 S9**

**Figure S7**. ^1^H NMR spectrum of compound **3 S10**

**Figure S8**. ^13^C NMR spectrum of compound **3 S11**

**Figure S9**. HPLC quantitative data of compound **3 S12**

**Figure S10**. ^1^H NMR spectrum of compound **4 S13**

**Figure S11**. ^13^C NMR spectrum of compound **4 S14**

**Figure S12**. HPLC quantitative data of compound **4 S15**

**Figure S13**. ^1^H NMR spectrum of compound **5 S16**

**Figure S14**. ^13^C NMR spectrum of compound **5 S17**

**Figure S15**. HPLC quantitative data of compound **5 S18**

**Figure S16**. ^1^H NMR spectrum of compound **6 S19**

**Figure S17**. ^13^C NMR spectrum of compound **6 S20**

**Figure S18**. HPLC quantitative data of compound **6 S21**

**Figure S19**. ^1^H NMR spectrum of compound **7 S22**

**Figure S20**. ^13^C NMR spectrum of compound **7 S23**

**Figure S21**. HPLC quantitative data of compound **7 S24**

**Figure S22**. ^1^H NMR spectrum of compound **8 S25**

**Figure S23**. HPLC quantitative data of compound **8 S26**

**Figure S24**. ^1^H NMR spectrum of compound **9 S27**

**Figure S25**. ^13^C NMR spectrum of compound **9 S28**

**Figure S26**. HPLC quantitative data of compound **9 S29**

**Figure S27**. HPLC chromatograms of NJ20, compound **1**, **2**, **3** and **4**. **S30**

**Figure S28**. HPLC chromatograms of NJ20, compound **5**, **6**, **7**, **8** and **9**. **S31**

**Figure S1**. ^1^H NMR spectrum of compound **1**

**Figure S2**. ^13^C NMR spectrum of compound **1**


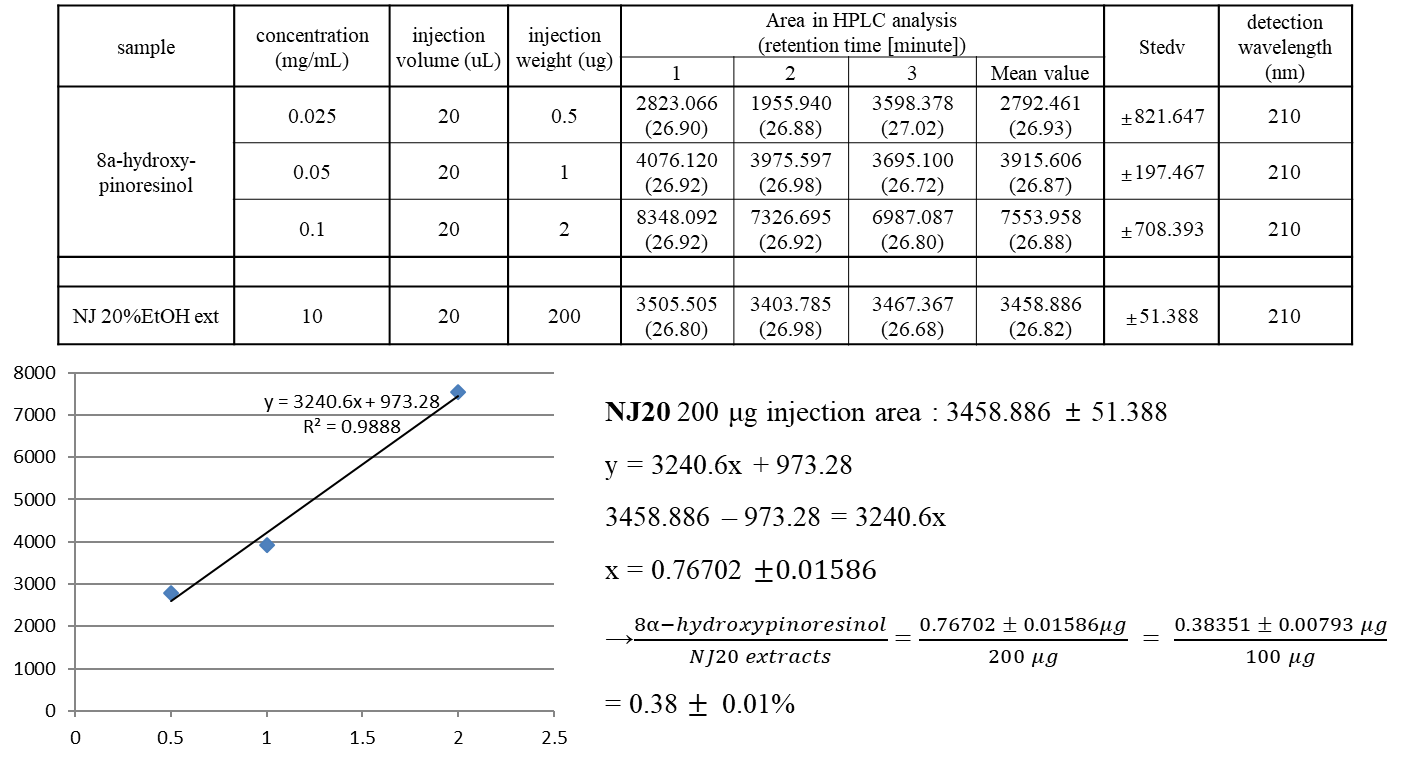


**Figure S3**. HPLC quantitative data of compound **1**

**Figure S4**. ^1^H NMR spectrum of compound **2**

**Figure S5**. ^13^C NMR spectrum of compound **2**


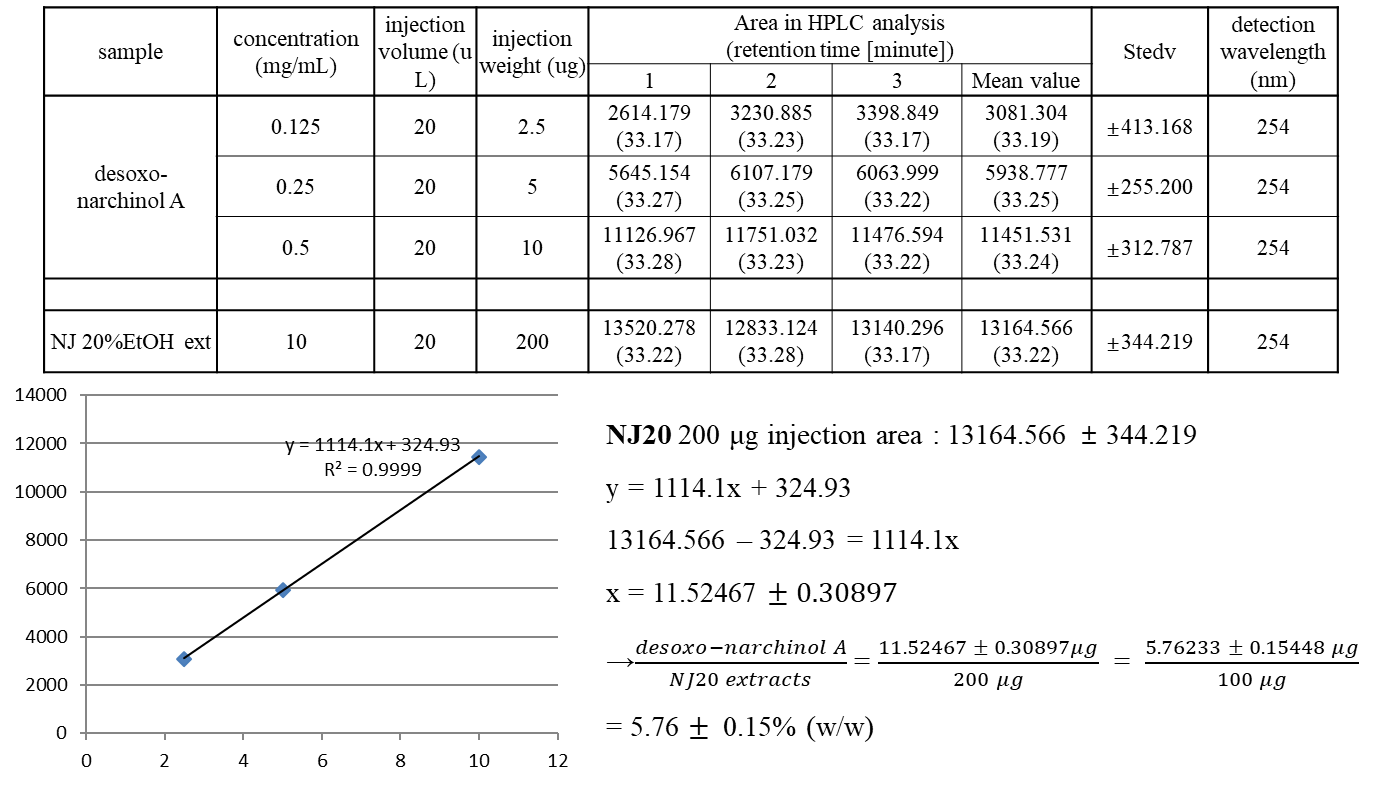


**Figure S6**. HPLC quantitative data of compound **2**

**Figure S7**. ^1^H NMR spectrum of compound **3**

**Figure S8**. ^13^C NMR spectrum of compound **3**


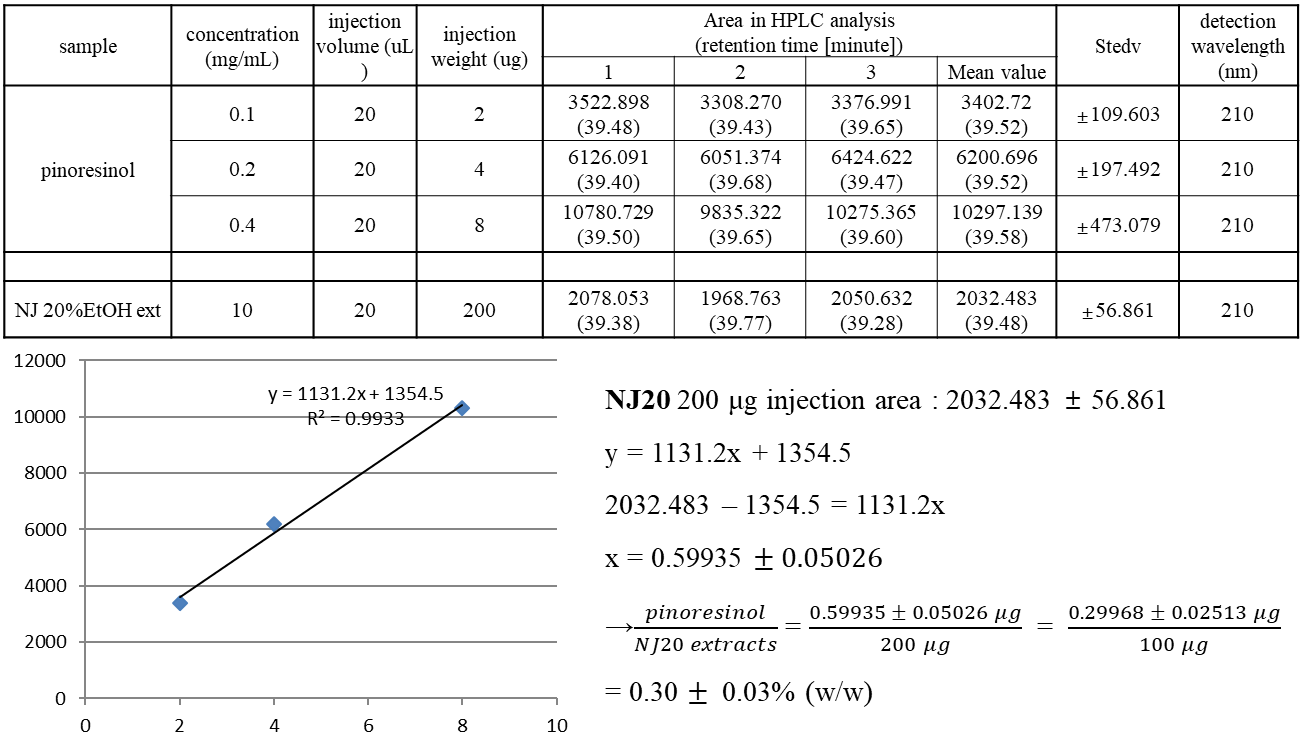


**Figure S9**. HPLC quantitative data of compound **3**

**Figure S10**. ^1^H NMR spectrum of compound **4**

**Figure S11**. ^13^C NMR spectrum of compound **4**

**
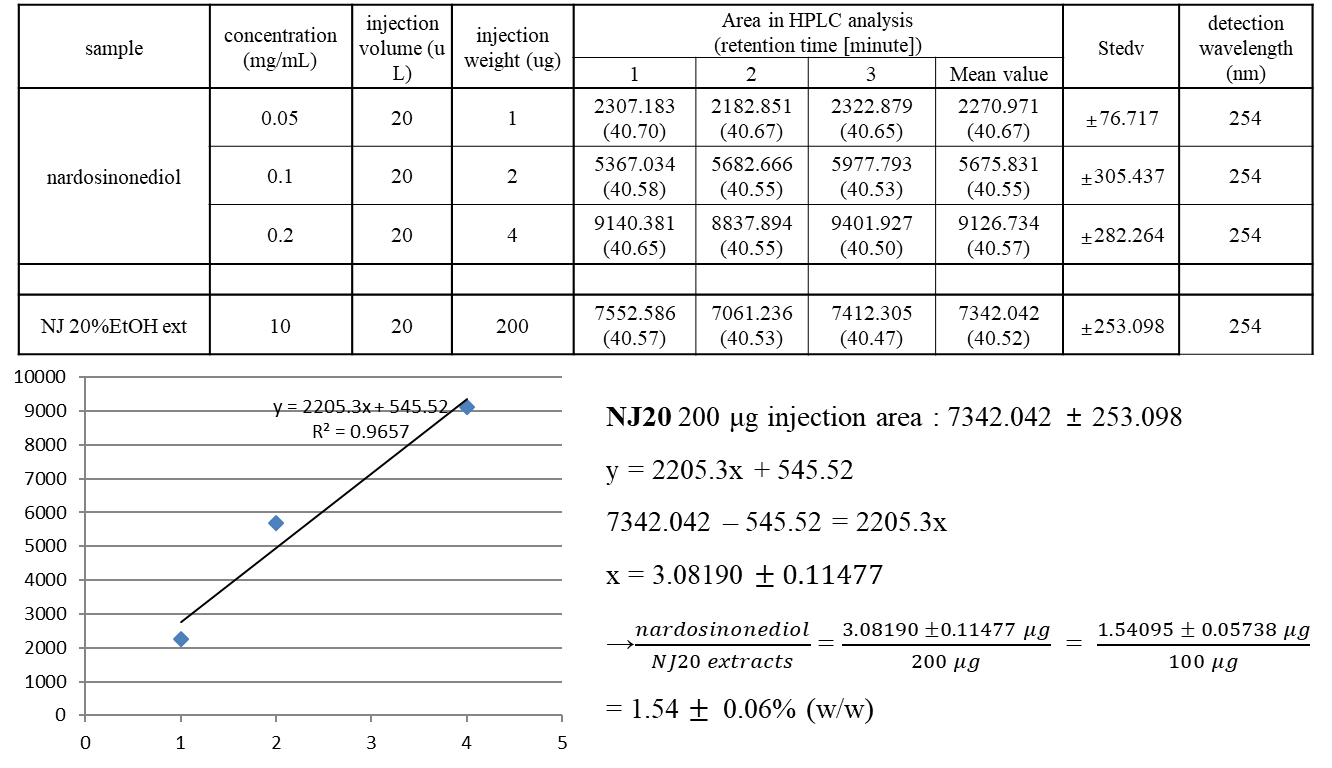
**

**Figure S12**. HPLC quantitative data of compound **4**

**Figure S13**. ^1^H NMR spectrum of compound **5**

**Figure S14**. ^13^C NMR spectrum of compound **5**


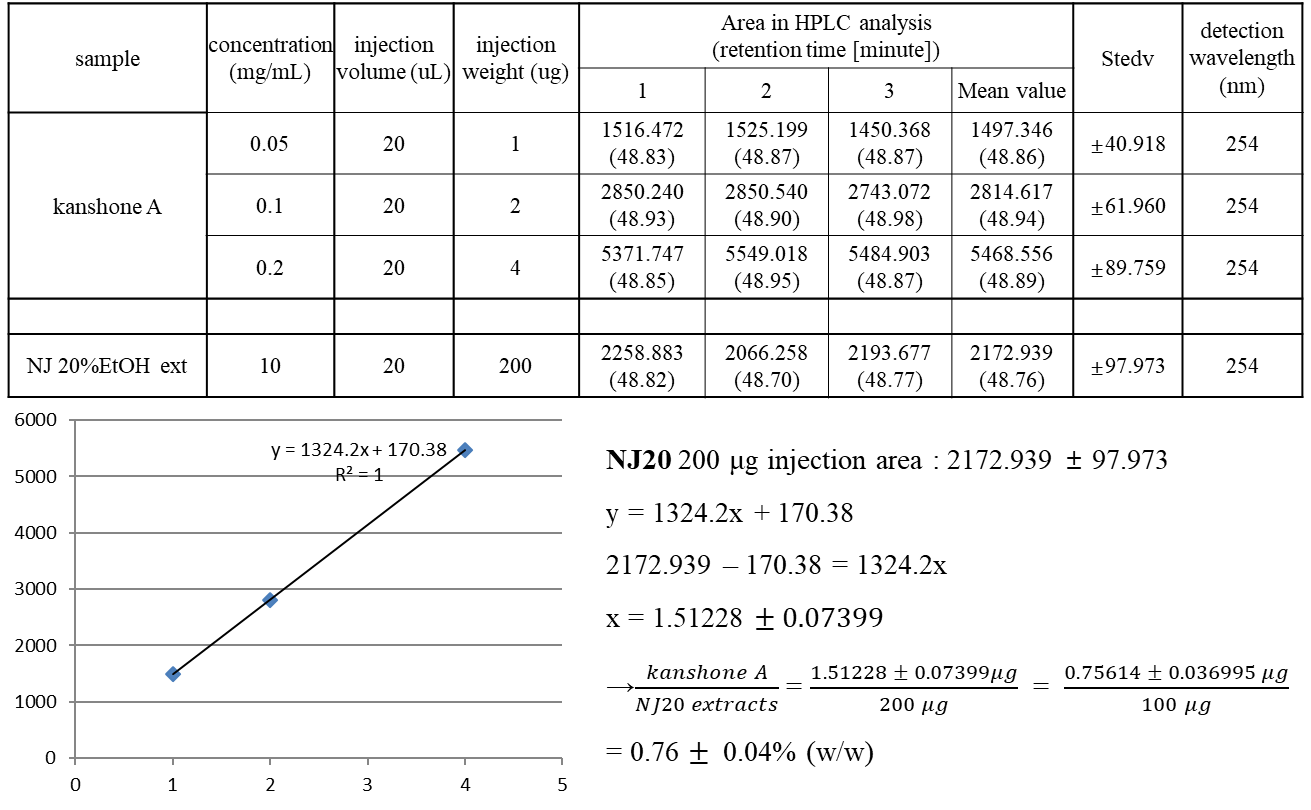


**Figure S15**. HPLC quantitative data of compound **5**

**Figure S16**. ^1^H NMR spectrum of compound **6**

**Figure S17**. ^13^C NMR spectrum of compound **6**


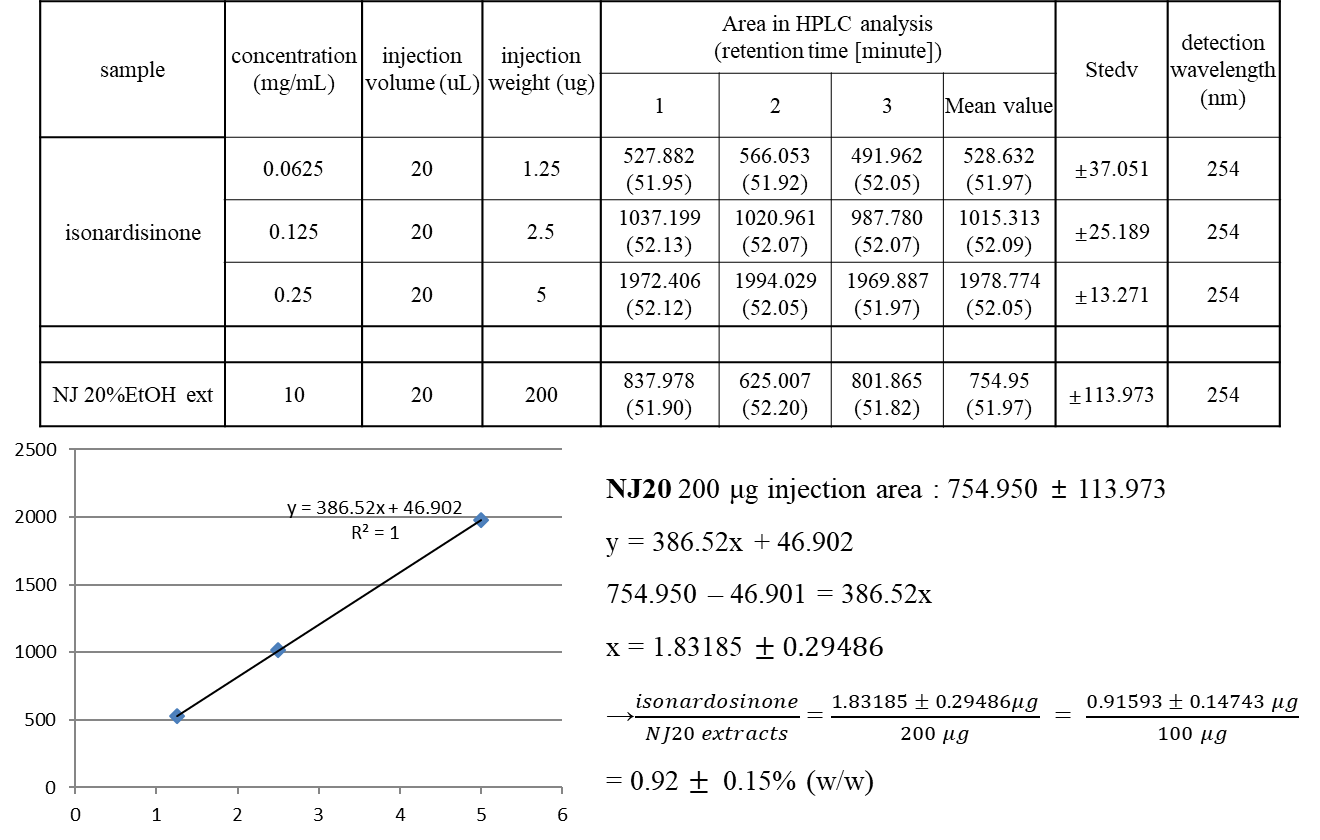


**Figure S18**. HPLC quantitative data of compound **6**

**Figure S19**. ^1^H NMR spectrum of compound **7**

**Figure S20**. ^13^C NMR spectrum of compound **7**


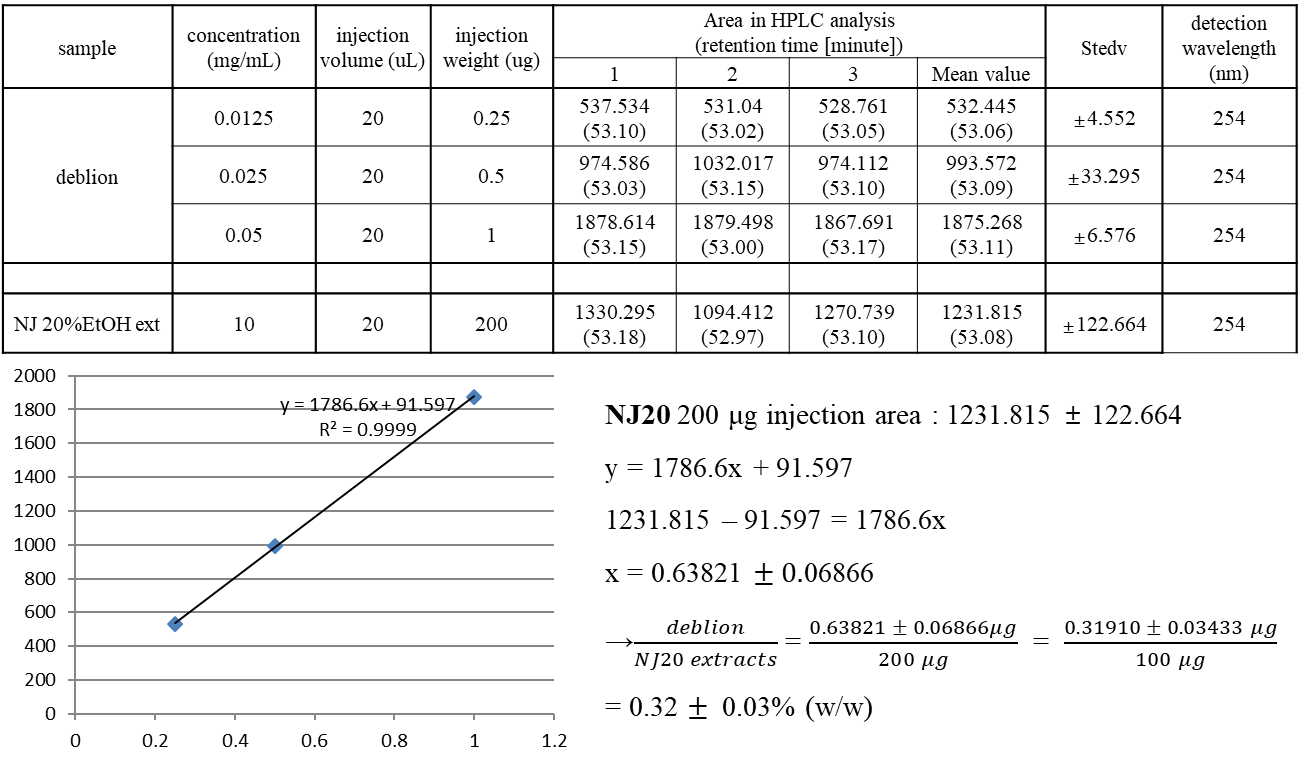


**Figure S21**. HPLC quantitative data of compound **7**

**Figure S22**. ^1^H NMR spectrum of compound **8**


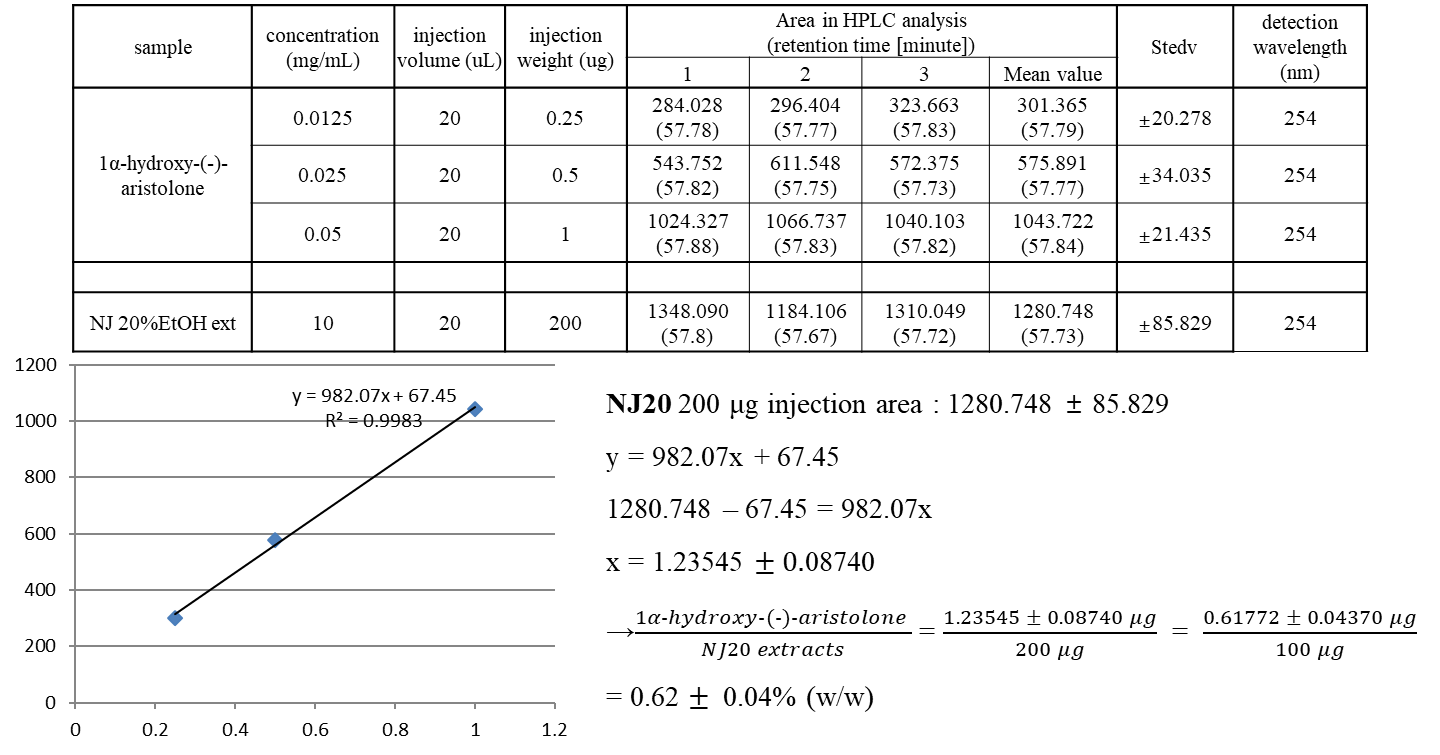


**Figure S23**. HPLC quantitative data of compound **8**

**Figure S24**. ^1^H NMR spectrum of compound **9**

**Figure S25**. ^13^C NMR spectrum of compound **9**


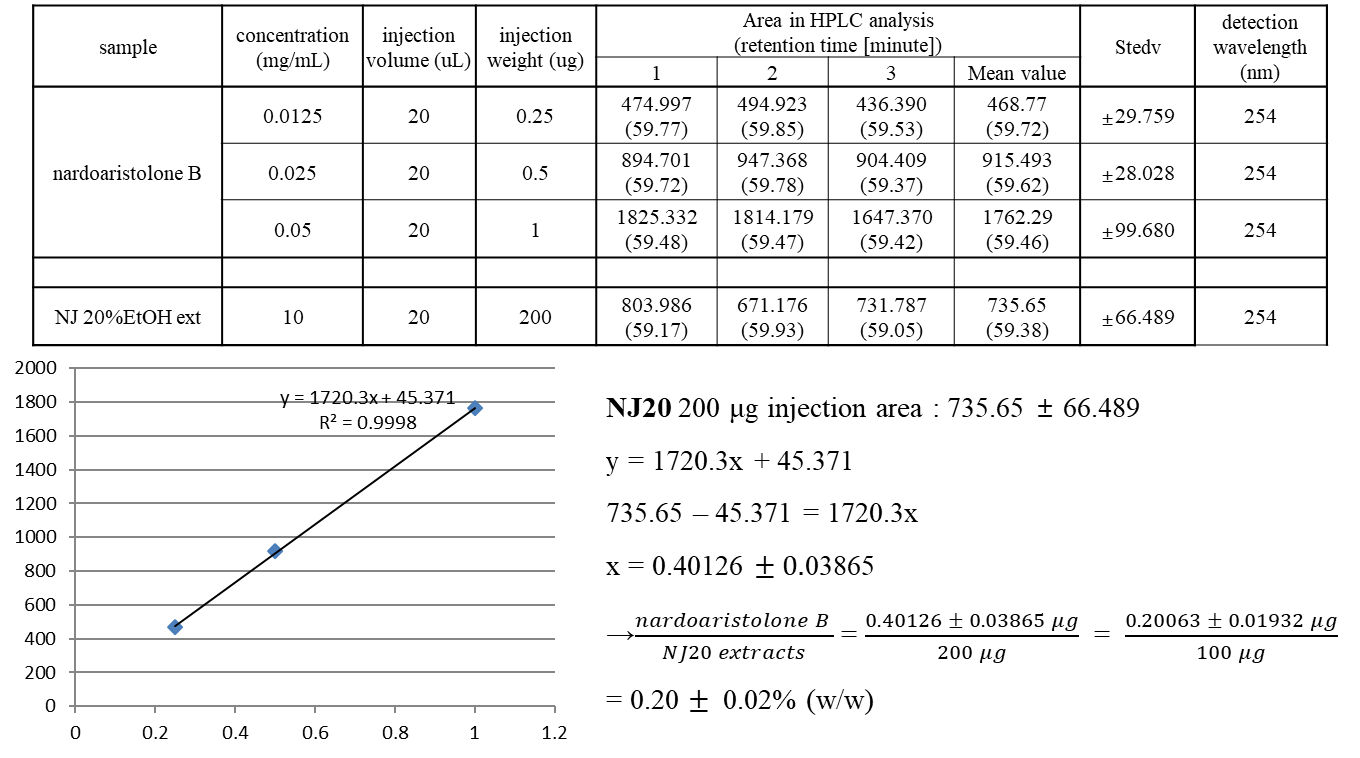


**Figure S26**. HPLC quantitative data of compound **9**


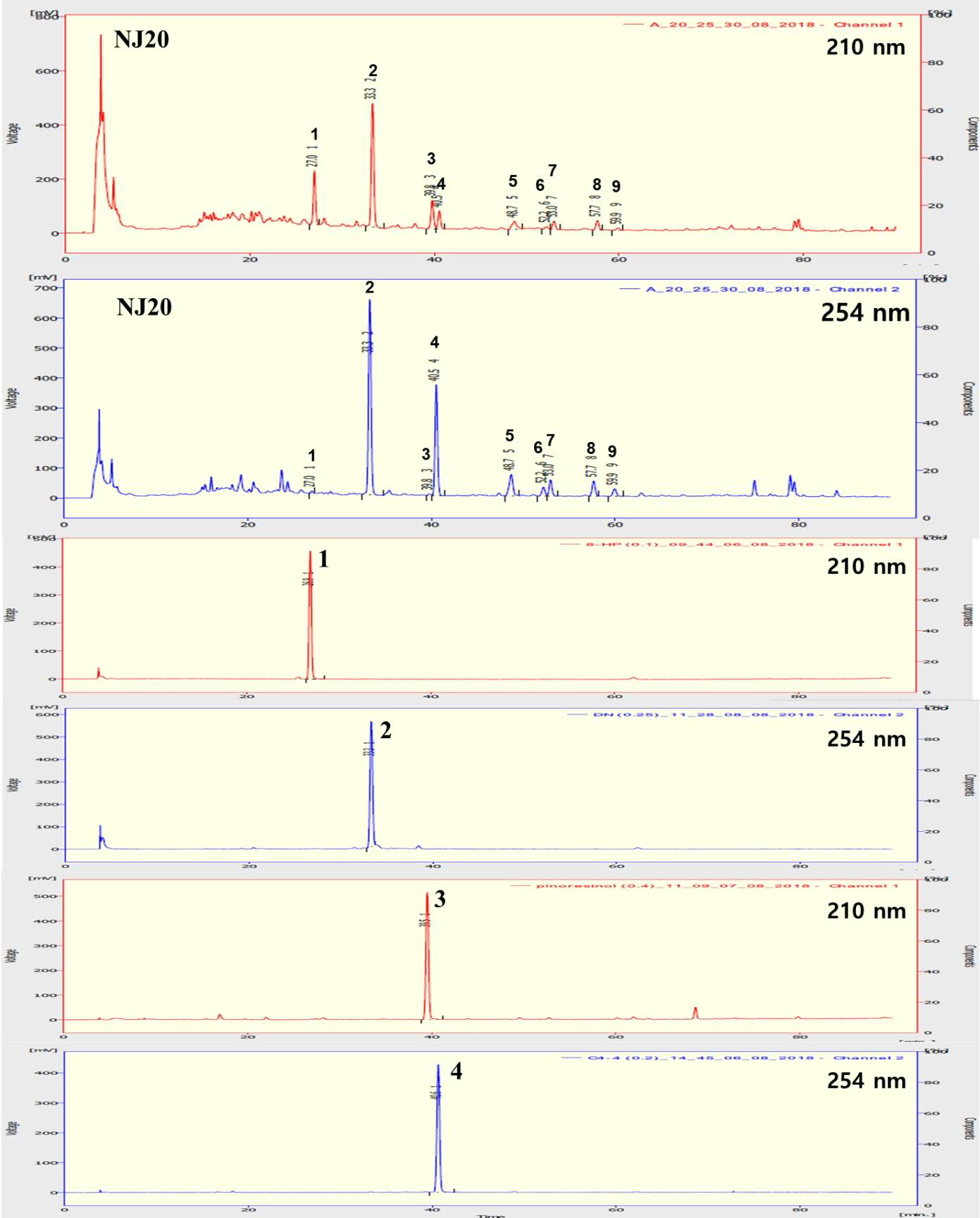


**Figure S27**. HPLC chromatograms of NJ20, compound **1**, **2**, **3** and **4**.


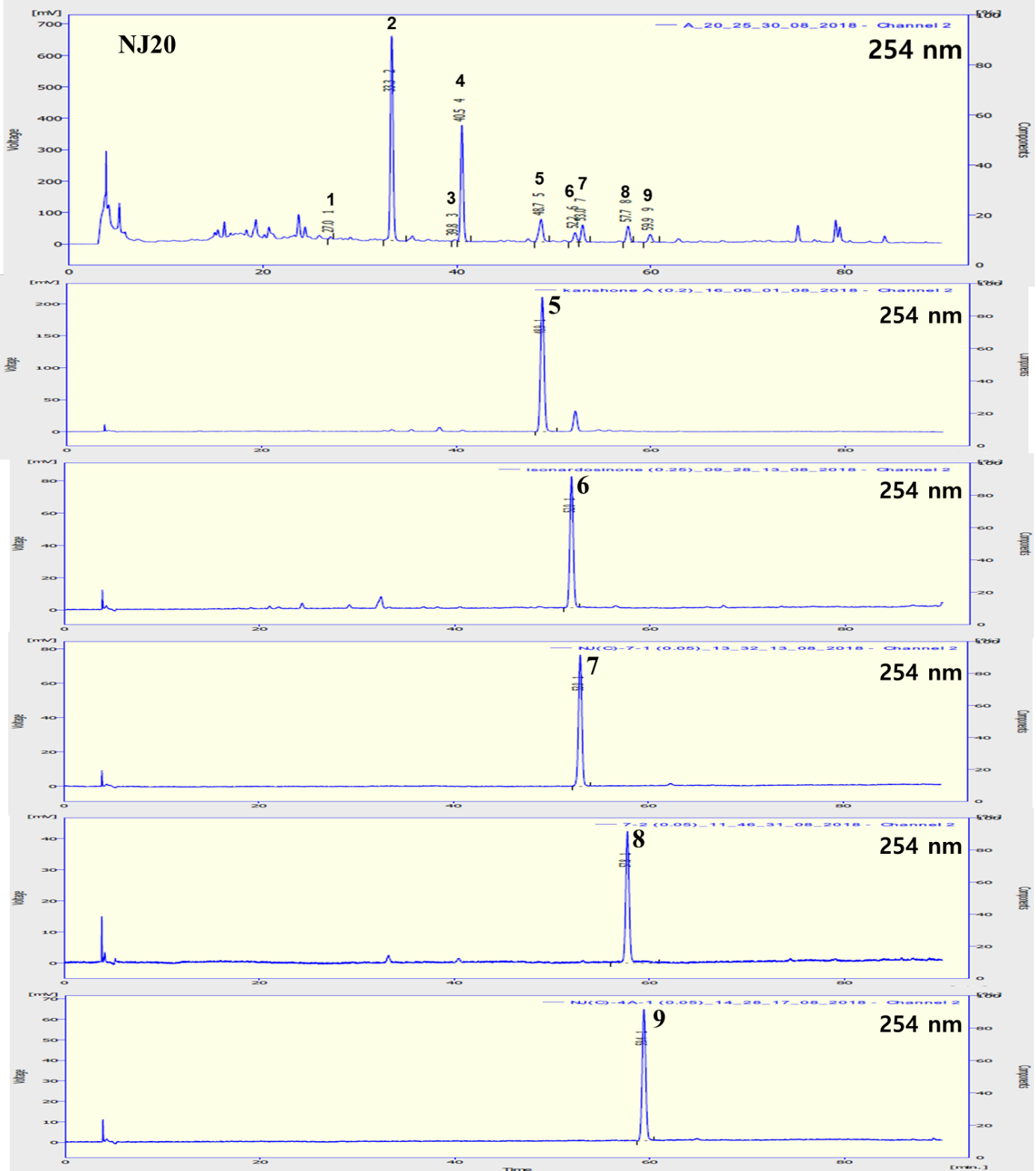


**Figure S28**. HPLC chromatograms of NJ20, compound **5**, **6**, **7**, **8** and **9**.
